# Supplementary material for: Spectrum of antibiotic resistant bacteria and fungi isolated from chronically infected wounds in a rural district hospital in Ghana
Source: PLoS One. 2020 Aug 7;15(8):e0237263. doi: 10.1371/journal.pone.0237263 (PMC7413558; doi:10.1371/journal.pone.0237263)
Supplement: S1 Table — (DOCX) [file pone.0237263.s001.docx]

**Table S1:** 207 Potential pathogenic bacteria isolated from 105 patient wounds.

| **Isolate** | **Frequency (%)** |
| --- | --- |
| ***Enterobacteriaceae***  *Klebsiella pneumoniae*  *Proteus mirabilis*  *Escherichia coli*  *Other Enterobacteriaceae* | 84 (41)  21 (10)  21 (10)  13 (6)  29 (14) |
| *Pseudomonas aeruginosa* | 50 (24) |
| *Staphylococcus aureus* | 28 (14) |
| *Enterococcus faecalis* | 12 (6) |
| *Streptococcus pyogenes* | 9 (4) |
| non-lactose fermenters | 8 (4) |
| *Enterococcus* spp. | 8 (4) |
| Other beta-haemolytic streptococci | 7 (3) |
