# Supplementary material for: Spectrum of antibiotic resistant bacteria and fungi isolated from chronically infected wounds in a rural district hospital in Ghana
Source: PLoS One. 2020 Aug 7;15(8):e0237263. doi: 10.1371/journal.pone.0237263 (PMC7413558; doi:10.1371/journal.pone.0237263)
Supplement: Attachment — Submitted filename: answer to reviewer_wounds_final.docx [file pone.0237263.s002.docx]

**Dear Editor and Reviewers,**

Thank you for the valuable input and suggestions. Please find below our comments in italics. We have amended the manuscript accordingly and do hope that the changes are acceptable to you.

Kind regards,

Denise Dekker

**Specific comments**

1. It is customary to abbreviate the name of the genus after the first time it is used, for examples, Staphylococcus aureus should be *S. aureus* after first mention in the paper (Ref:  International Code of Nomenclature of Bacteria: Bacteriological Code, 1990 Revision). ***Genus names were abbreviated accordingly.***
2. It may be valuable to have brief description of the different stages of wounds to give readers a general idea since this was mentioned in the results. ***Please refer to method for added description of different wound grades (lines 101-104). We also added how the wound was measured.***
3. Line 59 – 61:  Move this sentence down as third paragraph and expand to explain what other fungal pathogens could cause problem. ***Sentence was moved down a paragraph (73-78) and the paragraph was expanded.***
4. Line 72 – 73:  The rationale of the study needs to be strengthen further here.-***Please see additional information on the rationale of this study (lines 81 to 89).***
5. Line 87 – 90:  Need approval number-***this was added*** ***(line 110)***
6. Line 229 – 234:  This begs the question on how to access whether there are biofilm formation on the open wound. ***Discussion, second paragraph: We agree and admit that in this study, histological assessment of biofilms on chronic wounds was not performed. Accordingly, assumptions on the likely impact of biofilm formation remain speculative.***
7. Line 253 – 255:  This sentence is awkward, please rephrase for clarity. ***Sentence was changed- see now line 320-323***
8. Line 335 – 340:  Reference 9&10 need space in between for format consistency. ***Space was added***

**Reviewer 1**

9. It’s is not clear why Ghana rural localization was chosen for such observation. For me it is obvious, specially because I could observe some Ghanaian authors. However, at introduction authors are invited to better explain spot location, adding such information in a World context.- ***We have added information on the choice of study area. Please refer to lines 92-96***
10. In line 99, authors described that take 6 hours to transport the sample. I assume that collection was in a not easy place. But is this not a limitation? Authors could at least describe the possible contaminations occurring at this time. ***We have added information on this to “limitations of this study”. Please see lines 379-381.***
11. In line 113, authors are invited to better describe the MALDI biotyper analyses including the number of spectra used in each identification and the number of technical and biological replicates utilized- ***Information was added to lines 142-149.***
12. Please provide the list of antibiotics used for susceptibility tests. This could be added as supplementary material.- ***The list of antibiotics used and tested for each bacterial species are summarized in table 2. We stated the following L151-154 “Susceptibility to locally-available antibiotics was tested by the disk diffusion method and interpreted following the European Committee on Antimicrobial Susceptibility Testing (EUCAST) guidelines v.6.0 (http://www.eucast.org). Antibiotics of choice for bacterial isolates are illustrated in Table 2.***
13. Please provide the primers sequence used at fungal identification.- ***The primer sequence was added to line 166 & 167***
14. There is some problem in Figure 1. The same occurs in figure 2, inserted in main text. Nevertheless at the end of main text is everything ok. ***To us the figures seem fine. We are very happy to undertake changes whenever necessary. Please specify what needs changing.***
15. Table 2. It’s is amazing that all strains were 100% resistant to CXM. This is not impossible but unusual. Authors are invited to clearly discussed. Moreover, is also remarkable that Klebsiella show so high rates of resistance. This is also an important data. ***L327 & 330: a sentence was added to emphasize on the overall high level of resistance, also including second generation cephalosporins such as cefuroxime and a reference with similar data of Ghana.***
16. Why did authors did not test the fungal susceptibility to antifungals? Such data could also be important in order to determine the difficult in the region.-***This is a very important point. Unfortunately, it was not possible to culture for fungal isolates in the laboratory available on site hence antifungal susceptibility testing could not be carried out.***

**Reviewer 2:**

17. The aetiology of the chronic wounds in Table 1 is not welly defined (does cut means surgical incision or trauma?). Suggest for author to classify the types of wound by diabetic, arterial, infectious, surgical, trauma, venous and pressure ulcers. ***We do regret to not have obtained the etiology of the wound in detail, however most patients presenting with a chronic wound often cannot recall the initial incident which may have happened months before during e.g. hard practical work in the fields. Furthermore, testing for diabetes and for other aetiological causes were not performed regularly in this resource limited hospital as they were not covered by the national health insurance.***
18. Authors have performed logistic regression for wound size, traditional medicine and multiple bacterial infections. The p-value for the regression analysis are not stated in the manuscript. ***We calculated the risk ratio from 2x2-tables. We report confidence intervals (CIs) solely because, as also stated in the SAMPL Guidelines for Statistical Reporting which are applied by PLoS Journals, that CIs are preferred over p-values. Especially because of the exploratory nature of the analysis we would like to avoid to report results from null-hypothesis testing.***
19. Suggest to look into
    1. Association of antibiotic usage and antibiotic resistance.
    ***We appreciate this idea; however, the frequency of the respective isolates is too low to allow further analysis on the association with antibiotic usage.***
    2. Association of types of wound and antibiotic resistance.
    ***Again, the presence of particular bacteria in the respective wounds are too low to allow further analysis on the association with antibiotic resistance.***
20. In row 211, authors have mentioned that "In 23 (22%) wound, fungal DNA was amplified by broad range PCR". Does it means only 23 wounds were amplified for fungal detection instead of 105 wounds? ***Please see changes in row 293 and in heading of table 3.***

**Reviewer 3**

• Ethics Statement and Ethical considerations (lines 86-90): Please, include the approval number of Ethical Committee-***approval number was added.***
• Please, change “flora” with “microbiota” in all parts of the MS- ***The term flora was replaced by “microbiota” in all parts of MS.***
• Page 7, lines 122-123, please insert “ATCC” before the number of the strains-***ATCC was added before the number of the respective strain.***
• Page 7, line 150. Please, change 47 with 49- ***Please note that data from 47 patients was reported only. Two were missing.***
• Page 7 lines 160-163, Were the antimicrobial patterns of strains isolated from patients previously treated with antibiotics different from those of strains isolated from patients that used herbal treatments? Please insert a comment in Discussion ***– Unfortunately, the number of particular isolates are too low to estimate associations with herbal treatment.***
• Page 7, line 160. Please, add the space (n=11;10%)-***space was added***
• Page 12, line 228. Could be useful insert a comment regarding the following reference:
Synergistic interactions of Pseudomonas aeruginosa and Staphylococcus aureus in an in vitro wound model. DeLeon S, Clinton A, Fowler H, Everett J, Horswill AR, Rumbaugh KP. Infect Immun. 2014 Nov;82(11):4718-28. doi: 10.1128/IAI.02198-14. Epub 2014 Aug 25. ***We agree and have added a comment at the respective site.***
• Page 12, lines 235-237 Please the sentence is not clear, please rephrase it-***sentence was rephrased into one sentence-***
• Page 12, line 238. Please, add uppercase letter-***this was changed***
• Page 12, line 239 delete full stop and insert comma-***this was changed accordingly***
• Page 13, lines 255-260. Please, rephrase the sentences. ***Sentence was rephrased***
• Page 13, line 259 delete full stop and insert comma-***replaced***
• Page 16, line 305, change number 3 with 1***-changed***
• Figure 1 it is not clear, please, re-write the legend or modify the figure – ***Figure legend rewritten.***
